# Analysis of HIV quasispecies and virological outcome of an HIV D+/R+ kidney–liver transplantation

**DOI:** 10.1186/s12985-021-01730-w

**Published:** 2022-01-06

**Authors:** Gabriella Rozera, Ubaldo Visco-Comandini, Emanuela Giombini, Francesco Santini, Federica Forbici, Giulia Berno, Cesare Gruber, Paolo De Paolis, Roberto Colonnelli, Gianpiero D’Offizi, Giuseppe Maria Ettorre, Paolo Grossi, Maria Rosaria Capobianchi, Giuseppe Ippolito, Isabella Abbate

**Affiliations:** 1 Virology Unit, National Institute for Infectious Diseases, I.R.C.C.S. L.Spallanzani, Via Portuense, 292, 00149 Rome, Italy; 2 Hepatology Unit, P.O.I.T. San Camillo-Spallanzani, Rome, Italy; 3 Nefrology Unit, P.O.I.T. San Camillo-Spallanzani, Rome, Italy; 4 Surgical Unit, P.O.I.T. San Camillo-Spallanzani, Rome, Italy; 5 Insubria University, Varese, Italy; 6 Scientific Direction, National Institute for Infectious Diseases, I.R.C.C.S. L.Spallanzani, Rome, Italy

**Keywords:** HIV, Solid organ transplantation, Quasispecies, Viral reactivation

## Abstract

**Introduction:**

Transplantation among HIV positive patients may be a valuable therapeutic intervention. This study involves an HIV D+/R+ kidney–liver transplantation, where PBMC-associated HIV quasispecies were analyzed in donor and transplant recipients (TR) prior to transplantation and thereafter, together with standard viral monitoring.

**Methods:**

The donor was a 54 year of age HIV infected woman: kidney and liver recipients were two HIV infected men, aged 49 and 61. HIV quasispecies in PBMC was analyzed by ultra-deep sequencing of V3 *env* region. During TR follow-up, plasma HIV-1 RNA, HIV-1 DNA in PBMC, analysis of proviral integration sites and drug-resistance genotyping were performed. Other virological and immunological monitoring included CMV and EBV DNA quantification in blood and CD4 T cell counts.

**Results:**

Donor and TR were all ART-HIV suppressed at transplantation. Thereafter, TR maintained a nearly suppressed HIV-1 viremia, but HIV-1 RNA blips and the increase of proviral integration sites in PBMC attested some residual HIV replication. A transient peak in HIV-1 DNA occurred in the liver recipient. No major changes of drug-resistance genotype were detected after transplantation. CMV and EBV transient reactivations were observed only in the kidney recipient, but did not require specific treatment. CD4 counts remained stable. No intermixed quasispecies between donor and TR was observed at transplantation or thereafter. Despite signs of viral evolution in TR, HIV genetic heterogeneity did not increase over the course of the months of follow up.

**Conclusions:**

No evidence of HIV superinfection was observed in the donor nor in the recipients. The immunosuppressive treatment administrated to TR did not result in clinical relevant viral reactivations.

## Introduction

Kidney transplantation is a primary therapy for end-stage renal disease, just as orthotopic liver transplant (OLT) is considered to be the best curative treatment for patients with hepatocellular carcinoma (HCC) [1, 2]. HIV-positive individuals have a higher incidence of end-stage renal disease (ESRD) and face nearly a threefold higher mortality on dialysis, compared to their HIV-negative counterparts [3–6]. HCV/HIV or HBV/HIV co-infection are frequent in people who inject drugs (PWID) [7, 8]. HCC is a relevant cause of mortality in co-infected patients [9, 10], since HIV-related immunosuppression enhances viral replication in liver cells contributing to HCC pathogenesis [11]. Advances in combined antiviral therapy (ART) however, have made HIV infection a manageable chronic disease. People currently living with HIV and on ART have a near normal lifespan, and are suitable candidates to receive organ transplant, similar to the general population [12]. HIV+ donor to HIV+ recipient (HIV D+/R+) kidney transplantation was pioneered in South Africa in 2008 [13]. In Italy, HIV infected people became suitable organ donors for HIV positive recipients from 2018. Despite multicenter pilot studies reported that overall patient and graft survival in HIV+ donor to HIV+ recipients were excellent [13–15], the main concern about HIV/HIV transplantation is the possibility of donor derived HIV superinfection of the recipients. Kidneys and livers are considered a reservoir of HIV infection: compartimentalized HIV replication has been demonstrated in kidneys, with site-specific viral variants in urine segregating from those present in plasma [16, 17], whereas livers may harbor latently infected cells in subjects under effective antiviral treatment [18]. Ultra-deep sequencing (UDS) of viral quasispecies is a poweful tool to investigate variant mixture among infected individuals and has been used to trace transmission chains and cluster identification [19, 20].

The aim of this study was to analyse donor and recipients HIV quasispecies in a D+/R+ kidney–liver transplantation to highlight the possible donor-derived superinfection and to monitor any viral reactivations as well as their clinical consequences.

## Methods

### Study population

The organ donor was a 54-year-old HIV infected deceased woman who had been under suppressive ART since 1997. There was no evidence of viral failure (plasma HIV-1 RNA always under 200 cp/ml). Her treatment consisted in darunavir/cobicistat monotherapy with no other documented chronic active viral infections. The cause of death was a spontaneous brain hemorrhage*.* At the time of organ risk assessment for donation, HIV-1 RNA was not detected in plasma, CD4 count was 951 cells/mm^3^, with negative HBV/HCV markers.

The kidney recipient was a 49 year-old haemophilic patient with end stage renal disease on hemodialysis; he was infected with HIV (CDC stage B3), HBV (HBsAb+) and HCV (undetectable HCV-RNA). At the time of transplant, he had been under successfull ART (raltegravir plus rilpivirine) with HIV-1 RNA ≤ 50 copies/ml for almost 10 years. Previous ART included NRTIs, NNRTIs and protease inhibitors, with occasional HIV-1 RNA viral loads up to 800 copies/ml between 2003 and 2008. HIV drug resistance genotype performed on PBMC DNA at the time of transplant showed the presence of both NRTIs and NNRTIs associated resistance mutations (D67D/N, T69T/N, K70R, K103K/R, K219K/Q). The liver recipient was diagnosed with HCV related cirrhosis and untreatable hepatocellular carcinoma inside Milan criteria. HCV infection was succesfully treated with DAA in 2016. HBV markers were consistent with efficient immune control (HBcAb+, HBsAb). A good virologic control was obtained with the last ART (darunavir/cobicistat and raltegravir). After transplant, a new ART (emtricitabine/tenofovir alafenanide and dolutegravir 50 mg BID) based on the previous GRT results was initiated, to avoid drug-drug interactions with post-transplant anti-rejection therapy. HIV drug resistance genotype performed on PBMC DNA at the time of transplant highlighted the presence of only 3 secondary mutations in viral protease (L63P, V77I and I93L). Both recipients signed a written consent for the use of their clinical data and remaining biological samples for research activities. Authorization for use of clinical data and leftover biological samples for research is not needed by our Institute, according to the Italian Personal Data Protection Code (Legislative Decree No. 196 of 30 June 2003) as amended by Legislative Decree No. 101 of 10 August 2018, article 110-bis.

### Virological evaluation

HIV-1 RNA in plasma was measured by Aptima HIV-1 Quant assay (Hologic Inc. San Diego, CA USA). PBMC associated total HIV-1 DNA was quantified as in [21] with a limit of detection of 2.15 Log copies/million PBMC. HIV-1 *pol* genotyping was performed on PBMC, as previously described [22, 23]. CMV DNA, and EBV DNA quantifications were performed on whole blood while BKV DNA was monitored in plasma and urine by CMV ELITE MGB, EBV ELITE MGB and BKV ELITE MGB kits, respectively on the ELITe InGenius Instrument (ELITech Group S.p.A, Torino, Italia).

### Proviral HIV integration site analysis

Digestion with restriction enzymes of 10 µg of PBMC extracted DNA, ligation to double stranded DNA of a linker and semi-nested PCR using primers complementary to both the linker DNA and the long terminal repeat (LTR) end of the HIV provirus were described in [24]. UDS was performed with the shotgun approach by using the Ion Torrent S5 platform (Thermofisher Scientific, *Waltham*, MA, USA), following the manufacturer protocols. High-quality reads were mapped on HIV-1 reference sequence using BWA v.0.7.12 [10.1093/bioinformatics/btp324]; reads containing LTR sequence were aligned on an HIV-1 reference genome [NCBI Accession Number KO3455.1] and on the Human Reference Genome [GRCh38], discarding all reads that mapped on multiple sites with SAMTOOLS software v.1.3.1 [10.1093/bioinformatics/btp352].

### HIV *env* region UDS and phylogenetic analysis

*Env* region amplification was performed on PBMC DNA by nested PCR: the first and the second PCR were carried out with Platinum quality proofreading polymerase (Invitrogen, by Life Technologies, Monza, Italy). Both PCR were composed of 30 cycles (94 °C for 2 min, 94 °C for 15 s, annealing at 60 °C for 30 s, extension at 68 °C for 1 min or 30 s and final elongation at 68 °C for 5 min) with the primers described in [20]. Sequencing was performed with the amplicon approach on Ion S5 sequencer, following the manufacturer protocols. The reads were corrected with an *in-house* developed pipeline described in [20]. Quasispecies complexity of *env* region was evaluated by Shannon entropy, normalizing for the number of total variants identified in each sample as described in [25].

## Results and discussion

At the time of transplant (September 2019), the organ donor had no detectable HIV-1 RNA in plasma, while the kidney and liver recipients showed < 30 and 59 HIV-1 RNA copies/ml in plasma samples, respectively. During the follow-up period (September 2019-May 2021), recipients were monitored for viro-immunological parameters of HIV infection and for the major pathogens able to reactivate in TR.

For HIV infection monitoring, together with HIV-1 plasma viremia, PBMC-associated total HIV-1 DNA was evaluated. Quantitative determinations of CMV and EBV viremia in whole blood were performed at regular intervals in both recipients, while BKV DNA measure in plasma and urine was carried out in the kidney transplanted patient.

**Fig. 1:**
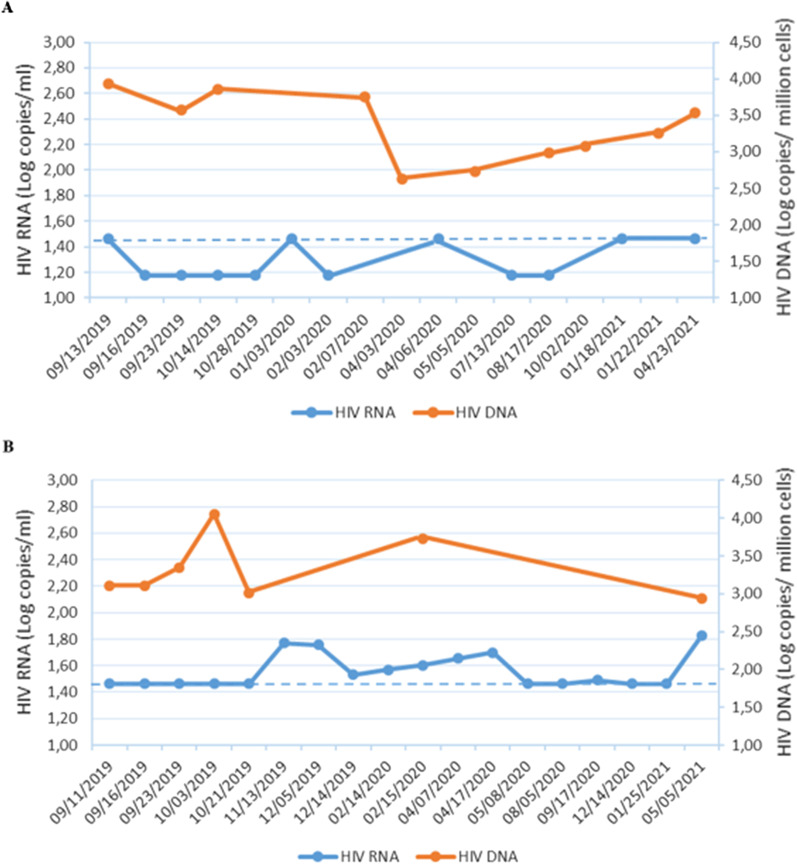
Kinetics of HIV-1 viremia and peripheral blood cellular reservoir of the infection in TR during the follow-up. Kinetics of HIV-1 RNA in plasma (blue line) and HIV-1 DNA in PBMC (red line) during the follow-up in kidney (**A**) and liver (**B**) recipients. The dotted line indicates the limit of quantification of HIV-1 RNA

In Fig. 1, the kinetics of HIV-1 RNA and PBMC-associated HIV-1 DNA in TR, starting from the time of transplant and throughout the whole follow-up period, are shown. In the kidney recipient, although plasma HIV-1 viremia always remained under the clinical threshold of 50 cp/ml, HIV-1 RNA was detected < 30 copies/ml at different time points; in the same period, the HIV cellular reservoir was almost stable (within < 0.5 Log copies/million cells) [median (IQR) 3.40 (2.92–3.78) copies/million cells]. The liver recipient invariably showed a detectable HIV-1 viremia with values above the clinical threshold of 50 copies/ml at different times and experienced transient peaks of HIV-1 DNA with values returning to basal levels at the last time of observation [median (IQR) 3.11 (3.01–3.73) copies/million cells].

CD4 T cell counts remained almost stable starting from the time of transplant and all throughout the follow-up period [median (IQR) 312 (240–354) and 272 (257–312) cell/mm^3^], in kidney and liver TR, respectively.

In order to provide additional evidence of residual HIV replication, proviral HIV integration site analysis was undertaken in PBMC collected at different times in both TR during the follow-up (Table 1). The reads containing the LTR region obtained for each sample, per time point, were median (IQR) 1858 (1113- 2466). In the kidney recipient (panel A) new integration sites were observed in serial samples together with an increase in the frequency of some integration sites, already detected at baseline; other integration sites present at baseline decreased in the frequency or were lost. In the liver recipient (panel B), the integration sites remained the same with almost identical frequencies, throughout the 5 months of follow-up.

**Table 1:** Proviral HIV integration sites in the PBMC of the kidney (A) and liver (B) recipients during the post-transplant period

Integration site (chromosome; position)	% of matches		
	09/13/2019	02/07/2020	01/22/2021
**A**			
NC_000015.10; 77401646	39.74	0	0
NC_000010.11; 21714841	29.23	0	0
NC_000005.10; 139967566	18.24	54.04	57.10
NC_000001.11; 226567704	3.04	0.11	0.20
NC_000002.12; 16097405	1.51	3.34	3.61
NC_000013.11; 59598085	1.28	0	9.37
NC_000011.10; 133588835	1.23	0	0
NC_000020.11; 56101682	0.37	0	0
NC_000013.11; 28414033	0.26	0	0
NC_000014.9; 52551000	0.24	0.54	0.41
NC_000020.11; 49687838	0.24	0.81	1.09
NC_000022.11; 46086683	0.15	0	0
NC_000004.12; 35007318	0.13	0	0
NC_000009.12; 122992409	0.05	0.54	0.08
NC_000009.12; 74259647	0	28.09	0
NC_000018.10; 36235516	0	1.35	0
NC_000004.12; 35007318	0	0.97	0.73
NC_000011.10; 58541534	0	0.81	0
NC_000007.14; 45947801	0	0.70	0
NC_000016.10; 5408445	0	0.48	0
NC_000016.10; 72345482	0	0.43	0
NC_000003.12; 163832295	0	0	9.61
NC_000001.11; 26998745	0	0	1.42
NC_000022.11; 46086683	0	0	0.93
NC_000017.11; 17716854	0	0	0.53
NC_000001.11; 196452109	0	0	0.45
NC_000016.10; 5408445	0	0	0.41
NC_000005.10; 140366572	0	0	0.41
NC_000001.11; 240464202	0	0	0.32

Integration site (chromosome; position)	% of matches		
	09/23/2019	02/15/2020	
**B**			
NC_000005.10; 139967566	87.96	87.64	
NC_000002.12; 16097405	4.31	4.53	
NC_000020.11; 49687838	1.62	1.65	
NC_000014.9; 52551000	1.26	0.82	
NC_000004.12; 35007318	1.17	1.03	

The integration sites are reported as human chromosome position; the frequencies for each integration site and for each time point evaluated were calculated as number of reads matched on that site, divided the number of total reads containing a LTR sequence obtained from that sample (% of matches). Only frequencies deriving from at least 8 matches were considered

In both recipients, drug resistance genotype performed on proviral DNA in PBMC did not change during the follow-up. The kidney recipient experienced a small EBV reactivation with a peak of 3,567 IU/ml of EBV DNA in blood soon after transplantation and a transient asymptomatic CMV reactivation with a peak value of 12,454 IU/ml CMV DNA in blood after 12 weeks, not requiring specific treatment. BKV DNA in urine and plasma remained undetectable for the entire follow-up period. The liver recipient did not show any CMV/EBV reactivations.

**Fig. 2:**
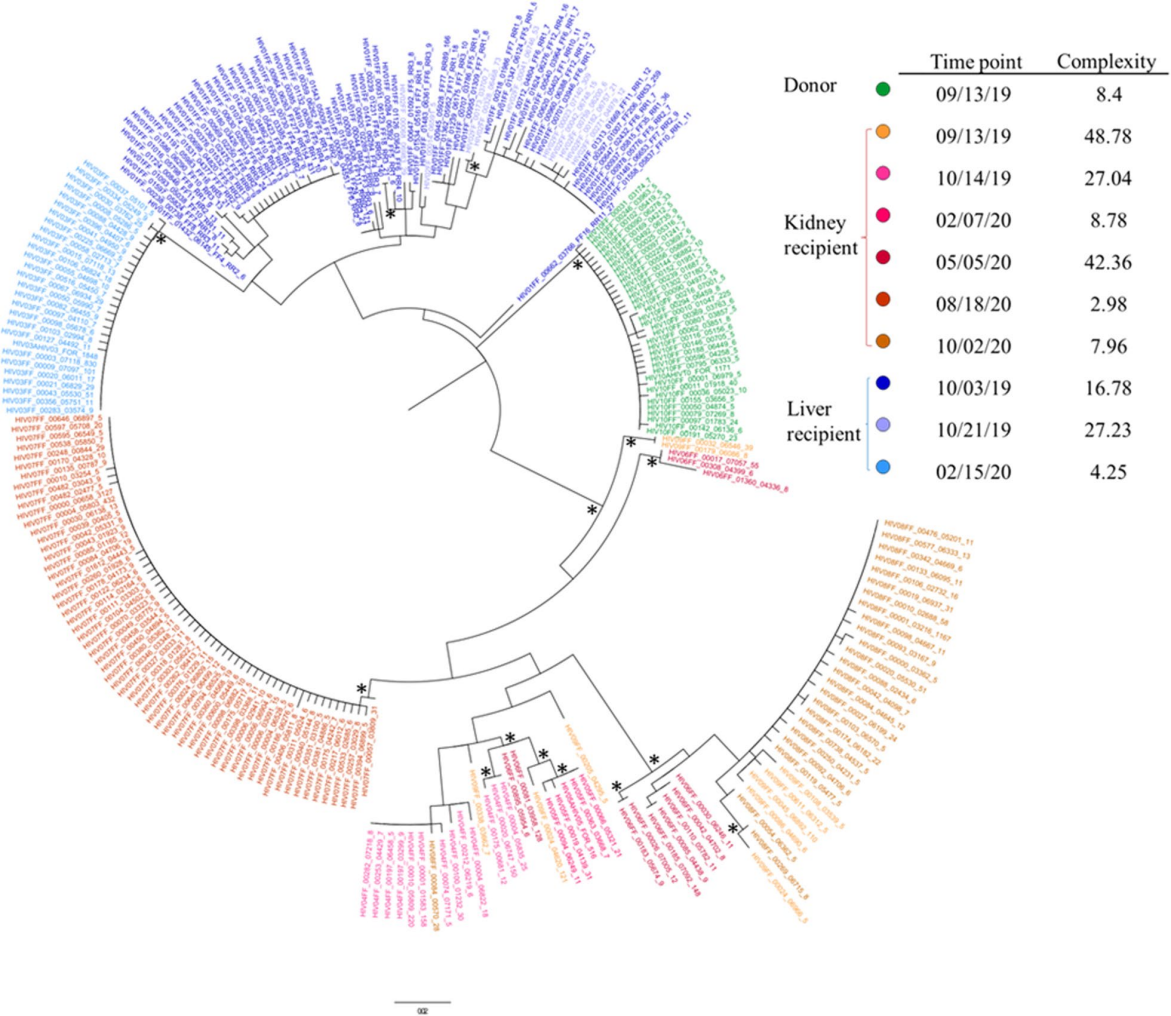
Phylogenetic tree of donor and recipients *env* sequences. Phylogenetic tree constructed with all the representative *env* sequences obtained from donor (green), kidney recipient (red/orange shades) and liver recipient (blue shades) at all time points. Bootstrap values > 85% were considered statistically significant (*). In the insert, complexity (normalized Shannon entropy) associated with each sample, from each patient, at the indicated time of collection, is shown

In order to highlight possible transmission of HIV variants from donor to recipients, an extensive phylogenetic analysis by ultra-deep sequencing of HIV *env* region was performed in donor and recipients at the time of transplant and in TR at different time points during the follow-up period (Fig. 2).

A median (IQR) of 1109 (511-2101) of *env* corrected sequences was obtained per patient/time point. Regarding viral tropism, all patients carried predominant R5 virus at transplantation and thereafter. The phylogenetic tree constructed with all the corrected sequences from both donor and recipients studied at different times, with respect to the time of transplant, showed complete segregation of HIV quasispecies between the subjects. This implied the absence of donor super-infection of the recipients and an independent genetic evolution in each TR during the follow-up, as suggested by the various sub-clustering observed among each recipient sequences over time. However, this was associated with a decrease of viral complexity overtime, in both TR (see insert in Fig. 2).

Despite the use of a powerful tool able to identify very low minority variants in the HIV quasispecies, this study did not observe any evidence of donor derived variants in TR, as was the case in the recent multicentre study [26]. Moreover, it has to be pointed out that previous studies, which highlighted HIV superinfection of recipients after HIV D+/R+ transplantation, mostly involved donors with detectable HIV-1 RNA in plasma [15, 27, 28]. In our case, the organ donor had no detectable HIV-1 RNA in circulation at the time of the organ explant, but it was not possible to rule out a priori the possibility of superinfection of the recipients through the transplanted organs, since both kidney and liver are organ reservoirs of HIV infection. In general, people with HIV superinfection have a less favourable prognosis, displaying lower CD4+ T-cell counts, higher viral loads and a shorter time to adverse clinical events, as compared to mono-infected persons [29, 30]. In addition, viral recombination in the cells of dually infected persons may occur, resulting in recombinant strains that could be resistant to antiretroviral therapies [31].

In our case, although phylogenetic analysis excluded donor derived superinfection, a low level of HIV replication persisted in both recipients, especially soon after transplantation. This was probably due to the temporary suspension of ART (2–3 days), during the stay in the intensive care unit after transplant. Indirect evidence of residual HIV replication during TR follow-up was the increase in the frequencies or the appearance of new specific types of integration sites, at least in one patient. Phylogenetic analysis also proved residual HIV replication, since in both TR some viral evolution was observed in the transplant follow-up. Immune-suppressive therapy, administrated to contrast organ transplant rejection, probably played a role in favouring HIV replication. It has been shown that persistent CMV and EBV shedding could contribute to the dynamics of the HIV-1 DNA reservoir during suppressive ART, increasing proviral genetic heterogeneity and HIV disease progression [32, 33]. In this study, CMV and EBV reactivations after transplantation were not associated with an increase of HIV heterogeneity, even in the presence of viral evolution, since in both recipients the HIV-1 DNA quasispecies complexity tended to decrease over time during follow-up.

## Conclusions

No evidence of HIV superinfection was observed in our donor/recipients couple, confirming that HIV D+/R+ transplantation may be an acceptably safe and valuable therapeutic intervention for HIV infected people. The immunosuppressive treatment provided to minimize transplant rejection, despite being able to promote HIV and other viral reactivations, does not seem to have had a major impact on HIV disease progression. Nevertheless, larger studies on these topics in the future could better consolidate the present conclusions.

## Data Availability

The datasets generated and analysed during the current study are available in the SRA database and will be accessible with the following link, https://www.ncbi.nlm.nih.gov/sra/PRJNA756451.

## Abbreviations

HIV: Human immunodeficiency virus
TR: Transplant recipients
PBMC: Peripheral blood mononuclear cell
CMV: Cytomegalovirus
EBV: Epstein–Barr virus
BKV: Polyomavirus BK
OLT: Orthotopic liver transplant
HCC: Hepatocellular carcinoma
HCV: Hepatitis C virus
HBV: Hepatitis B virus
ESRD: End-stage renal disease
PWID: People who inject drugs
ART: Antiretroviral therapy
NRTIs: Nucleoside reverse transcriptase inhibitor
NNRTIs: Non nucleoside reverse transcriptase inhibitor
DAA: Direct-acting antivirals
GRT: Genotypic resistance testing
HIV D+/R+: HIV+ donor to HIV+ recipient
UDS: Ultra-deep sequencing
LTR: Long terminal repeat
